# Exploiting domain transformation and deep learning for hand gesture recognition using a low-cost dataglove

**DOI:** 10.1038/s41598-022-25108-2

**Published:** 2022-12-12

**Authors:** Md. Ahasan Atick Faisal, Farhan Fuad Abir, Mosabber Uddin Ahmed, Md Atiqur Rahman Ahad

**Affiliations:** 1 Department of Electrical and Electronic Engineering, University of Dhaka, Dhaka, 1000 Bangladesh; 2 Department of Computer Science and Digital Technologies, University of East London, London, UK

**Keywords:** Biomedical engineering, Electrical and electronic engineering, Information technology

## Abstract

Hand gesture recognition is one of the most widely explored areas under the human–computer interaction domain. Although various modalities of hand gesture recognition have been explored in the last three decades, in recent years, due to the availability of hardware and deep learning algorithms, hand gesture recognition research has attained renewed momentum. In this paper, we evaluate the effectiveness of a low-cost dataglove for classifying hand gestures in the light of deep learning. We have developed a cost-effective dataglove using five flex sensors, an inertial measurement unit, and a powerful microcontroller for onboard processing and wireless connectivity. We have collected data from 25 subjects for 24 static and 16 dynamic American sign language gestures for validating our system. Moreover, we proposed a novel Spatial Projection Image-based technique for dynamic hand gesture recognition. We also explored a parallel-path neural network architecture for handling multimodal data more effectively. Our method produced an F1-score of 82.19% for static gestures and 97.35% for dynamic gestures from a leave-one-out-cross-validation approach. Overall, this study demonstrates the promising performance of a generalized hand gesture recognition technique in hand gesture recognition. The dataset used in this work has been made publicly available.

## Introduction

From the dawn of human civilization, communication between humans has been the single most important trait for our survival. At the same time, it created the social attributes among us which had been modified over the centuries and transformed us into civilized beings. However, the first mode of communication was not a structured vocal language but involved gestures, often using hands. Later, with the progress of civilization, people adopted structured languages and used hand gesture-based communication in special cases. Recent researchers have found around 6700 spoken languages^1^ and several hundred sign languages^2^, although a good number of them are not currently in use. Despite having structured vocal languages in every country, sign languages are still used primarily for communication with the deaf and hard-of-hearing community. However, since non-signers are not generally familiar with these sign languages, deaf and hard-of-hearing people face communication barriers. On the other hand, with the technological development in sensor technologies, embedded systems, camera technologies, and efficient learning systems, hand gesture recognition research has found efficient and pragmatic solutions to the communication problem.

Hand gestures and their use vary greatly depending on the field of application. Apart from sign-language communication, several other tasks, namely military coordination, interaction with digital devices, and virtual gaming consoles involve hand gestures. Based on hand motion, hand gestures can be divided into two types—static hand gestures and dynamic ones. Moreover, based on the application, one or both hands can be involved to complete a sign. Over the year, researchers have been trying to develop several technologies to use human hand gestures to communicate with the cyber world. Hence, the task of hand gesture recognition has been one of the most widely explored areas in the research domain of Human–Computer Interaction (HCI). The detection systems can be classified into two categories—contactless detection systems where the detecting device is kept at a distance from the hand and are not in any sort of contact, and wearable detection systems that are often implemented with several sensors at close contact with the hand^3^.

The researchers have explored contactless hand gesture recognition systems using several modalities, namely Radio Frequency (RF), ultrasound, and Computer Vision (CV). Google’s Project Soli is a 60 GHz millimeter-wave radar on-chip that can detect fine-grained hand gestures along with micro-finger movement^4^. Wang et al. used this chip and employed a deep learning algorithm to detect 11 dynamic hand gestures from 10 subjects^5^. However, this chip is not usable for detecting gestures at a meter-long distance. In this regard, WiFi has been used as a ubiquitous modality for detecting hand gestures from a greater distance than the Soli chip^6–8^; however, it fails in the precision of detection^3^. On the other hand, several studies have discovered the potential of ultrasound for detecting hand gestures with a clear line of sight^9–11^. Although in recent years, these RF-based and sound-based modalities have been improved in performance and reliability in particular applications, they are still not dependable in a regular use case where the environmental parameters vary frequently^3^.

Owing to the tremendous development in Artificial Intelligence (AI) and camera technology, computer vision-based gesture detection systems are the most widely explored field of research in recent years. Although fundamentally employing computer vision modality, hand tracking and gesture recognition can be achieved in a variety of techniques namely, skin color detection^12–14^, appearance detection^15–18^, motion-based detection^19,20^, skeleton-based detection^21,22^, and depth detection^23–26^. Apart from the conventional RGB camera, IR-based leap motion controller^27,28^ and Microsoft Kinect depth camera^26,29–31^ are two of the most widely used hardware for depth and skeletal information detection. However, the primary shortcomings of these methods are the environmental dynamics, namely lighting conditions, line of sight, and detector proximity. Although depth-based and skeleton-based approaches have become more robust over the year, such as the MediaPipe by Google^32^, they still have not overcome those shortcomings completely. Moreover, due to the cost of the high-quality depth sensor, the usability of such systems is rather still limited.

On the other hand, sensor-based wearable datagloves are one of the most widely used contact-based hand gesture recognition systems that have overcome most of the shortcomings of contactless detection methods. VPL Inc. first introduced a commercial sensor-based dataglove back in 1987^33^. The researcher of this dataglove invented optical flex sensors which enabled them to track finger flexion. Despite inventing the technology at such an early age of HCI, these datagloves were not widely adoptable due to the high cost and lack of feasibility in regular use. In the last decade, owing to the development of low-cost high-performance sensors, processing devices, connectivity, and algorithms, researchers have explored new avenues of sensor-based hand gesture recognition.

Over the year, a wide range of both commercially available and custom-made sensors are used on datagloves for accurately capturing hand gesture dynamics. Several studies have explored surface electromyography (sEMG) sensors to capture the electrical activity inside the hand muscles during gesture performance^34–40^. Moreover, Wang et al.^41^, Abreu et al.^42^, and Su et al.^43^ used variants of the Myo band which are commercial sEMG armbands and are specifically designed to track hand gestures. Although sEMG shows reliable performance in a wide range of gestures including sign languages, the detection algorithms were subjected to the sensor placement and the signers. Moreover, several studies have used resistive flex sensors and their variants for actively tracking finger flexions^44–46^ and an Inertial Measurement Unit (IMU) to detect the hand movements^40,44,45,47,48^. Wen et al. developed a smart glove with 15 triboelectric nanogenerator (TENG)-based sensors for tracking 50 American Sign Language (ASL) words and 20 sentences^49^. Furthermore, using a fusion of multiple sensors has shown greater performance in several studies than a single sensor method^40,41,43,45,46,50^.

In our previous study, we presented a dataglove with five flex sensors and one IMU and evaluated its performance in a limited number of gestures and subjects^44^. In this work, we adopted the same sensor fusion configuration and developed and combined it with state-of-the-art deep learning techniques. We proposed a Spatial Projection Image based deep learning technique for dynamic hand gesture recognition and parallel-path neural network architecture for multimodal sensor data analysis. The system successfully recognizes 40 words from the ASL dictionary, including 24 static and 16 dynamic signs collected from 25 subjects. In a nutshell, the key contributions of this work are as follows:

- We constructed a low-cost wireless capable dataglove combining flex sensors and IMU and explored state-of-the-art deep learning techniques on it.
- We provided a large dataset of 40 ASL letters and words collected from 25 subjects using the proposed dataglove.
- We introduced Spatial Projection to convert the 1D time-series signals into 2D images for dynamic gesture recognition which outperformed 1D CNN and classical machine learning-based approaches.
- The proposed parallel-path neural network architecture showed superior feature extraction capability from multimodal data over conventional architectures.

## Methods and materials

### Hardware configuration

**Figure 1:**
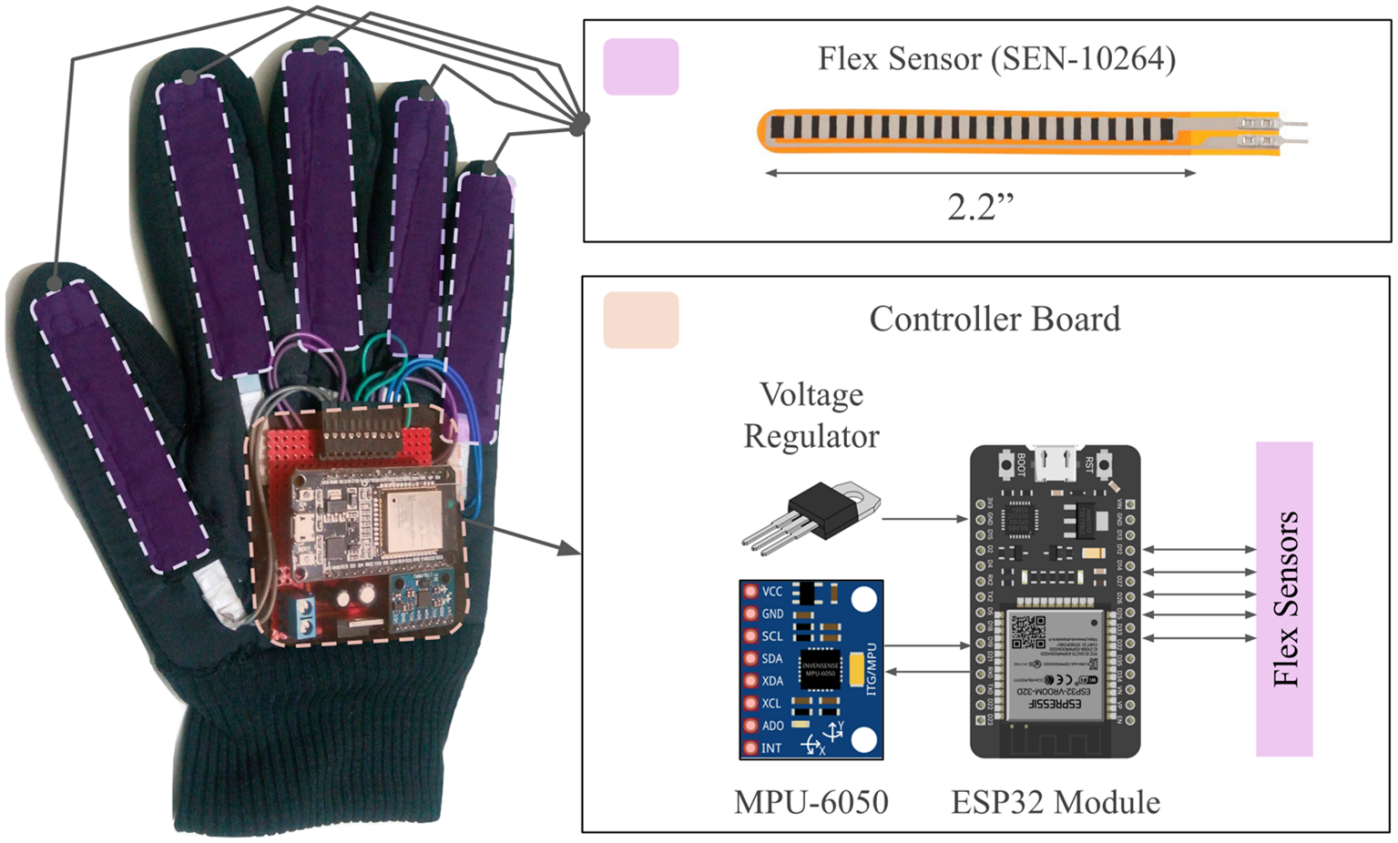
The dataglove architecture: On the left, we have the glove with all the mounted sensors and electronics. A flex sensor is shown in the top right corner. The components of the main controller board are shown in the bottom right corner. It consists of an ESP32 microcontroller, an MPU-6050 IMU, and some complementary electronics.

The primary hardware is a dataglove consisting of three units, namely sensing, processing, and onboard power regulation unit. The sensing unit is comprised of five 2.2" flex sensors (SEN-10264) and an IMU (MPU-6050) which has a triaxial accelerometer and a triaxial gyroscope. The overall hardware configuration is illustrated in Fig. 1.

#### Sensing unit

The flex sensors are, in fact, variable resistors with flat resistance of $25\;{\text{K}}\Omega \;\left( { \pm \;30\% } \right)$, which are placed above the five fingers of the dataglove using fabric pockets to sense the fingers’ flex. A voltage divider was created with each flex sensor and a $0.25 \;{\text{W}}\;100$ KΩ ($\pm \;5\% )$ resistor was used to convert the resistance difference during the finger flexion to the voltage difference across the sensor using the processing unit^51^.

The accelerometer and gyroscope of the IMU are configured to track the linear acceleration within $\pm \;19.6 \;{\text{ms}}^{ - 2}$ and angular velocity within $\pm \;4.36\;{\text{rad}}\;{\text{s}}^{ - 1}$, respectively, which is well within the range of any human hand motion. Moreover, the IMU contains a Digital Motion Processor (DMP) which can derive the quaternions in-chip from the accelerometers and gyroscope data and thus, provides the hand orientation data along with the motion information^52^.

#### Processing unit

The processing unit is a WiFi-enabled development module called DOIT ESP32 Devkit V1 that has a Tensilica Xtensa LX microprocessor with a maximum clock frequency of $240\;{\text{MHz}}$. The 12–bit analog to digital converter (ADC) with 200-kilo samples per second maximum sampling rate is capable of sampling the flex sensors’ analog data with sufficient resolution. Moreover, the module is capable of communicating with external computers via USB which enables wired data communication^53^.

#### Onboard power regulation

The ESP32 module and the IMU have an operating voltage of $3.3\;{\text{V}}$^52,53^. On the other hand, the flex sensors do not have a strict operating voltage^51^. Hence, we used an LM1117 low-dropout (LDO) 3.3 V linear voltage regulator to regulate the supply voltage from the $3.7\;{\text{V}}$ single cell LiPo battery. Moreover, we used $10$ ﻿μF and $100$ μF filtering capacitors to filter out the supply noise.

### Dataset

#### Overview

We explored 40 signs from the standard ASL dictionary that including 26 letters and 14 words. Among these signs, 24 require only a certain finger flexion and no hand motion; hence, are addressed as static signs or gestures. Conversely, the remaining 16 signs need hand motion alongside finger flexion to portray meaningful expression according to the ASL dictionary. Moreover, we collected the signs from 25 subjects (19 Male and 6 Female) in separate data recording sessions with a consistent protocol. Overall, three channels for acceleration in both body and earth axis, three for angular velocity, four for quaternion, and five for flex sensors were recorded in the dataset.

The data was recorded by the dataglove processing unit which was connected to a laptop for data storage via USB. The sampling frequency is set to 100 Hz and each gesture was repeated 10 times to record the performance variabilities of each subject. However, during a few sessions denoted in the dataset supplementary information, the laptop charger was connected which resulted in AC-induced noise all over those specific recorded data.

#### Data recording protocol

Before starting the recording process, each subject signed an approval form for the usage of their data in this research and was briefed about the data recording steps. As the subjects were not familiar with the signs before the study, they were taught each sign before the data recording via online video materials^54^. The data was recorded by the dataglove and stored on the laptop at the same time. Hence, a Python script was used on the laptop to make the handshake between the two devices and to store the data in separate folders as per the signs and the subjects.

**Figure 2:**
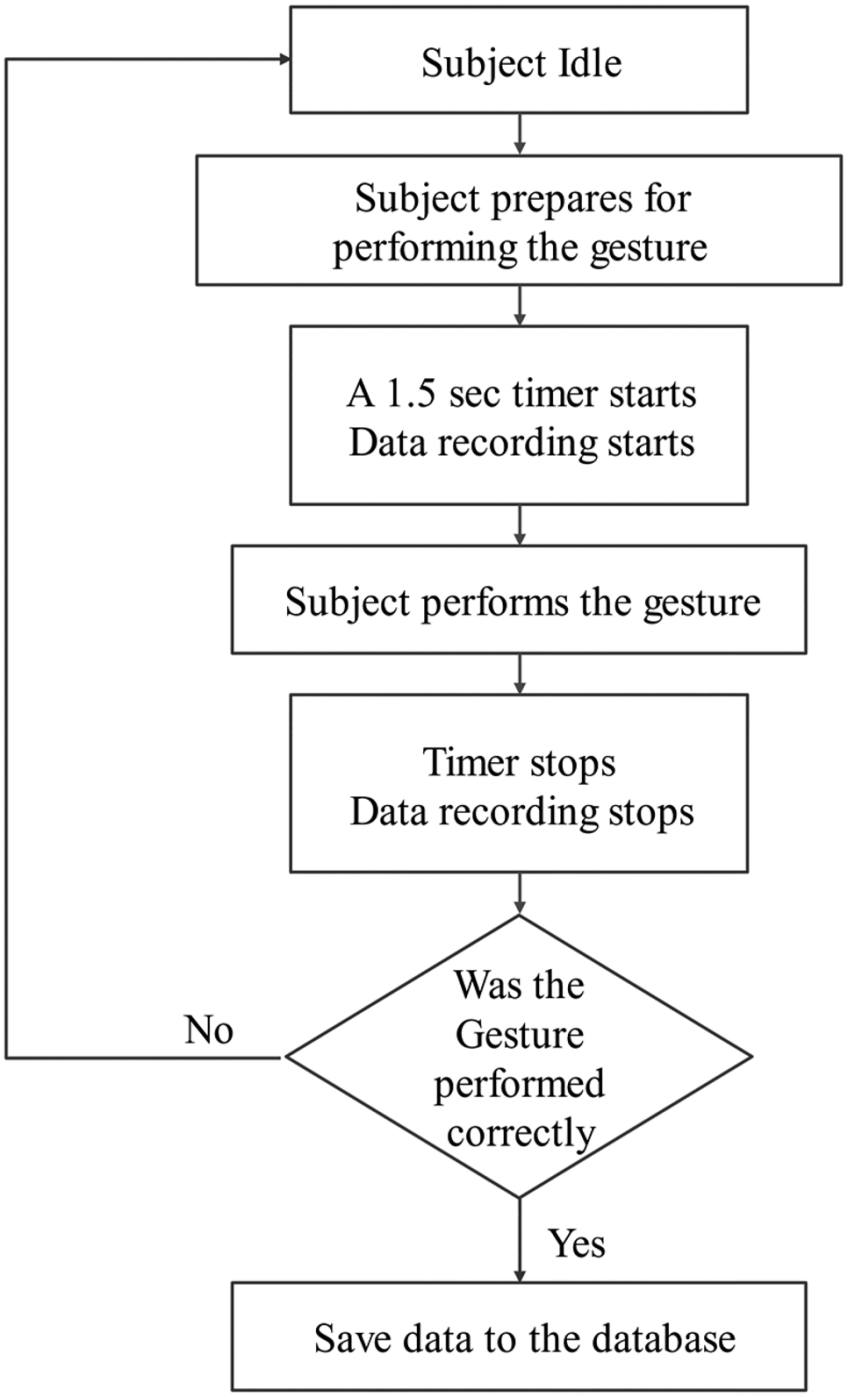
The flowchart showing the data collection protocol. The diagram shows all the different steps of the data collection process. This protocol was followed during the data collection for all the subjects.

At the beginning of each data recording session, the subjects were prompted to declare their subject id and the gesture name. Afterward, a five-second countdown is prompted on the laptop screen for preparation. Each instance of the gesture data is recorded for a 1.5 s window and the subjects can easily perform their gesture once within that window. In a single gesture recording session, this process is repeated 10 times. The gesture recording flow for each session is shown in Fig. 2. All methods were carried out following the relevant guidelines, and the correctness of gestures was evaluated by visual inspection. All experimental protocols were approved by the University of Dhaka, Dhaka, Bangladesh. Note that informed consent was obtained from all subjects.

### Data preprocessing

#### Gravity compensation

The triaxial accelerometer of the IMU body records acceleration, which is subjected to gravity. Hence, the gravity component has to be adjusted from the recorded raw acceleration to interpret the actual motion characteristics of the dataglove. The gravity vector can be derived from the orientation of the dataglove. Quaternions express the 3d orientation of an object which is a robust alternative to the Euler angles which are often affected by gimbal-lock^55^. The digital motion processor (DMP) of the MPU-6050 processes the raw acceleration and angular velocity internally and produces quaternion. The quaternions can be expressed by Eq. (1).

1 $${\varvec{Q}} = q_{w} + {\mathbf{q}} = q_{w} + q_{x} \hat{i} + q_{y} \hat{j} + q_{z} \hat{k}$$

where ${\varvec{Q}}$ stands for a quaternion that contains a scaler, ${q}_{w}$ and a vector, $\mathbf{q}\left({q}_{x},{q}_{y},{q}_{z}\right)$. The overall gravity compensation process is described in Eqs. (2) and (3)^56^.

2 $$\left[ {\begin{array}{*{20}c} {g_{x} } \\ {g_{y} } \\ {g_{z} } \\ \end{array} } \right] = \left\| g \right\|\left[ {\begin{array}{*{20}c} {2(q_{x} q_{z} - q_{w} q_{y} )} \\ {2(q_{w} q_{x} + q_{y} q_{z} )} \\ {q_{w}^{2} - q_{x}^{2} - q_{y}^{2} + q_{z}^{2} } \\ \end{array} } \right]$$

3 $$\left[ {\begin{array}{*{20}c} {la_{x} } \\ {la_{y} } \\ {la_{z} } \\ \end{array} } \right] = \left[ {\begin{array}{*{20}c} {a_{x} } \\ {a_{y} } \\ {a_{z} } \\ \end{array} } \right] - \left[ {\begin{array}{*{20}c} {g_{x} } \\ {g_{y} } \\ {g_{z} } \\ \end{array} } \right]$$

where ${\varvec{g}}\left({g}_{x},{g}_{y},{g}_{z}\right)$, ${\varvec{Q}}\left({q}_{w}, {q}_{x},{q}_{y},{q}_{z}\right),$ ${\varvec{l}}{\varvec{a}}\left({la}_{x},{la}_{y},{la}_{z}\right)$, and ${\varvec{a}}\left({a}_{x},{a}_{y},{a}_{z}\right)$ denotes the gravity vector, quaternion, linear acceleration vector, and raw acceleration vector, respectively. The resultant linear acceleration (${\varvec{l}}{\varvec{a}}$) represents the body axis acceleration which is compensated for the gravity offset. This step was done in the processing unit of the dataglove.

#### Axis rotation

**Figure 3:**
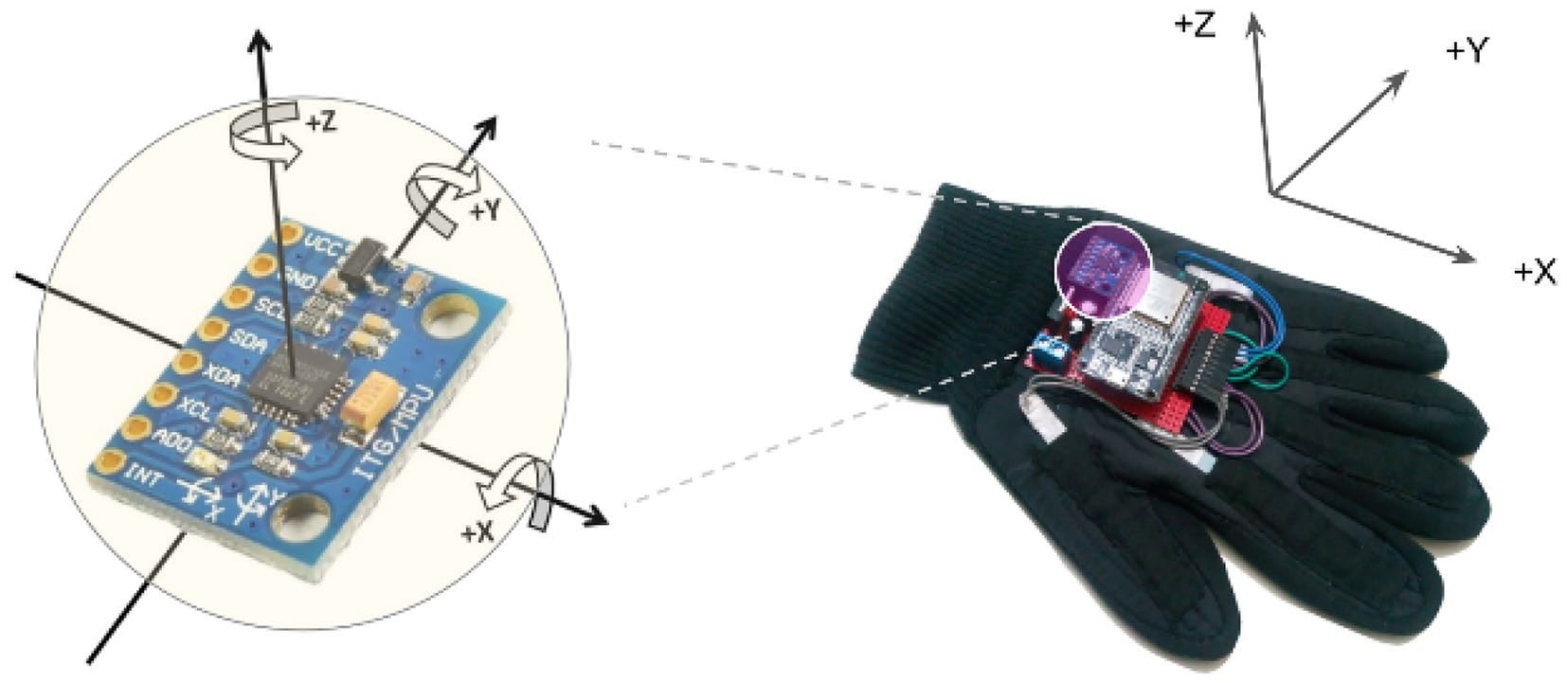
The IMU orientation diagram: On left, we have the X, Y, and Z coordinates of the MPU-6050. Along these 3 axes, the accelerometer and gyroscope values are recorded. The figure on the right shows the body axis to earth axis conversion diagram.

The recorded raw acceleration and the gravity-compensated linear acceleration both were in the body axis of the dataglove and the body axis is dependent on the initial orientation of the dataglove when it powers up. However, this nature of axis dependency on the initial orientation is problematic for real-world applications. Hence, we converted the triaxial acceleration vector from the body axis to the North-East-Down (NED) coordinate system which follows the directions based on the earth itself^57^. At first, a rotation matrix was calculated using the quaternions. Afterward, the NED linear acceleration is derived using matrix multiplication between the rotation matrix and the body axis linear acceleration. Equations (4) and (5) show this axis transformation process using quaternions^58^.

4 $${\mathbf{R = }}\left[ {\begin{array}{*{20}c} {1 - 2(q_{y}^{2} + q_{z}^{2} )} & {2(q_{x} q_{y} - q_{w} q_{z} )} & {2(q_{x} q_{z} - q_{w} q_{y} )} \\ {2(q_{x} q_{y} + q_{w} q_{z} )} & {1 - 2(q_{x}^{2} + q_{z}^{2} )} & {2(q_{y} q_{z} - q_{w} q_{x} )} \\ {2(q_{x} q_{z} + q_{w} q_{y} )} & {2(q_{y} q_{z} + q_{w} q_{x} )} & {1 - 2(q_{x}^{2} + q_{y}^{2} )} \\ \end{array} } \right]$$

5 $$\left[ {\begin{array}{*{20}c} {LA_{x} } \\ {LA_{y} } \\ {LA_{z} } \\ \end{array} } \right] = {\mathbf{R}}\left[ {\begin{array}{*{20}c} {la_{x} } \\ {la_{y} } \\ {la_{z} } \\ \end{array} } \right]$$

where $\mathbf{R}$, ${\varvec{Q}}\left({q}_{w}, {q}_{x},{q}_{y},{q}_{z}\right)$, ${\varvec{L}}{\varvec{A}}\left({LA}_{x},{LA}_{y},{LA}_{z}\right)$, and ${\varvec{l}}{\varvec{a}}\left({la}_{x},{la}_{y},{la}_{z}\right)$ stands for the rotation matrix, quaternion, NED linear acceleration, and the body axis linear acceleration, respectively. Similar to the previous step, this axis transformation is also done in the processing unit of the dataglove. Figure 3 illustrates the axial diagram of the dataglove and the axis rotation.

#### Rolling filters

After closer inspection, we found a few random spikes in the IMU data. Hence, firstly, we removed using a rolling median filter of 10 data points to get rid of such spikes. After the spike removal, secondly, we used an extra step of applying moving average filters for the only specific sessions where the recordings were subjected to AC-induced noise which resulted in comparable waveforms for all data recordings. The implementation of the moving average filter is shown in Eq. (6)^59^:

6 $$y\left[ n \right] = \frac{1}{N}\mathop \sum \limits_{k = 0}^{N - 1} x\left[ {n - k} \right]$$

where $x\left[n\right]$ is the input signal, $N$ stands for the number of data points, and $y\left[n\right]$ denotes the output signal. However, after applying the rolling average there were a few null values at the end of each signal frame which were replaced by the nearest values in that signal. According to the data recording protocol, the gestures were performed in the middle of each 1.5-s window. Hence, replacing the few terminal data points with the nearest available valid data point does not change the signal morphology. Lastly, we used another level of rolling average filter of 10 data points, this time for the whole dataset, to further smooth the signal and also replaced the terminal null values with the nearest valid data point in each frame.

#### Normalization

The processed acceleration and flex sensor data are not in the same range. Hence, before employing the AI-based classification technique, data normalization is widely practiced for better convergence of the loss function^60^. We used min–max scaling as the normalization technique with a range of $\left[ {0,1} \right]$. It is shown in Eq. (7)^61^:

7 $$x_{normalized} = \frac{{x - x_{\min } }}{{x_{\max } - x_{\min } }}$$

where $x$ is the input and ${x}_{normalized}$ is the normalized output. ${x}_{\mathrm{max}}$ and ${x}_{\mathrm{min}}$ respectively denote the maximum and minimum values of the input.

#### Spatial projection images generation

There are several challenges associated with dynamic sign language recognition. In our case, the temporal dependency and the size of the hand were the most challenging issues. A signer can perform a sign at many different speeds. Moreover, the speed does not match up from signer to signer. To successfully recognize signs from all the subjects, first, this temporal dependency needs to be removed from the signals. The second challenge was the hand size of the signer which introduced variability in the gestures performed by different signers. In the proposed method, we tried to eliminate these two issues by utilizing the Spatial Projection Images of the dynamic gestures. However, the static gestures do not generate a meaningful pattern in the projections due to their stationary nature. Hence, this step is omitted for static signs.

When interpreting a sign, the speed of performing the sign and the signer's hand size does not matter. The spatial pattern created by the motion of the signer’s hand defines the sign. As long as the pattern is correct, the sign will be considered valid regardless of its temporal and spatial states. To capture this pattern of sign language gestures we utilized the accelerometer sensor data from our device. Using Eqs. (8–9), we converted the 3D acceleration into 3D displacement vectors. These vectors represent the path followed by the hand in 3D space during the performance of the gesture.

8 $$\mathop \smallint \limits_{{t_{1} }}^{{t_{2} }} a\left( t \right)dt = v\left( {t_{2} ) - v(t_{1} } \right)$$

9 $$\mathop \smallint \limits_{{t_{1} }}^{{t_{2} }} v\left( t \right)dt = x\left( {t_{2} ) - x(t_{1} } \right)$$

**Figure 4:**
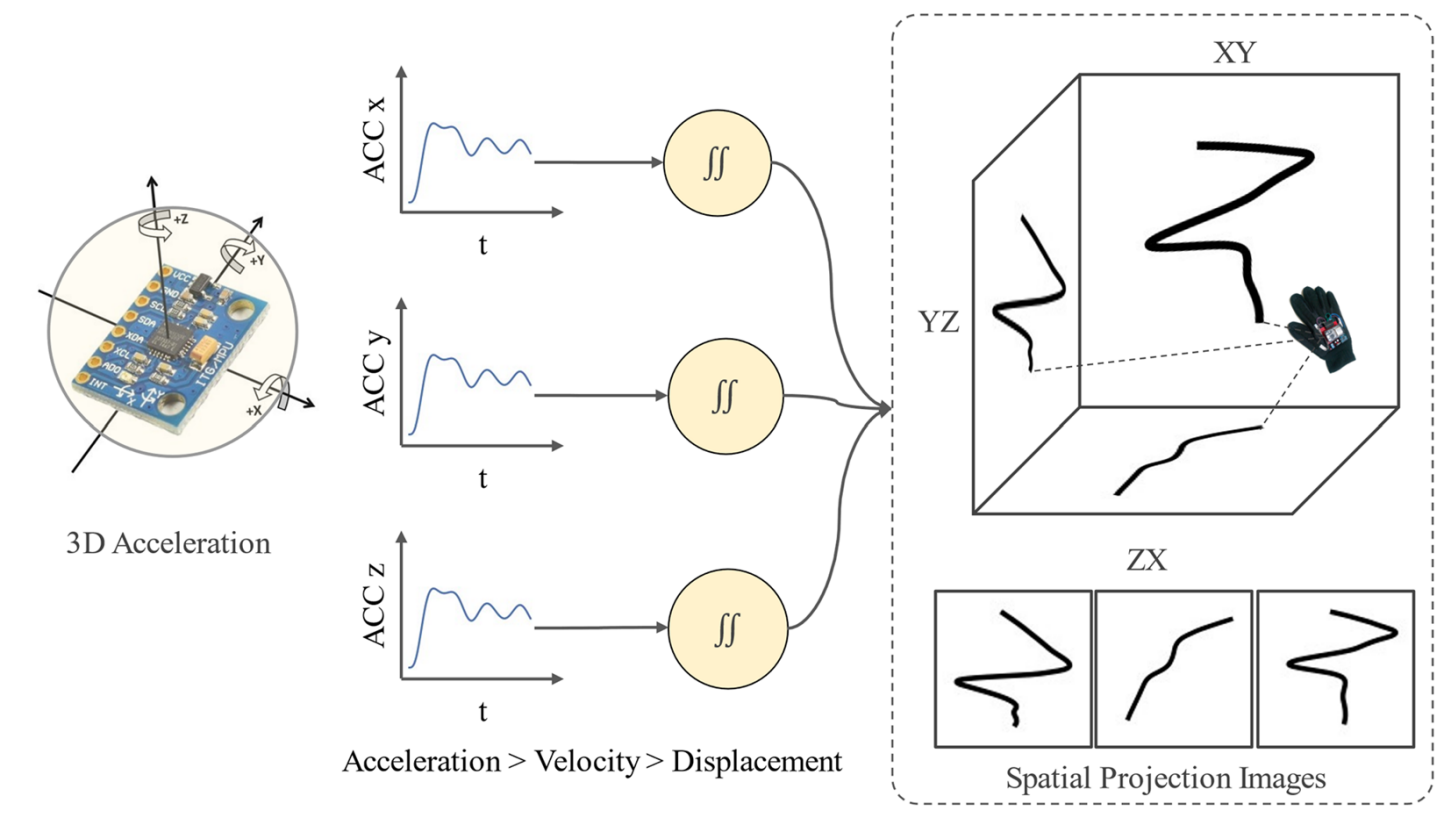
Spatial projection generation process. We start with the 3-axis acceleration and then convert them into 3-axis displacement vectors. These vectors are projected onto the 2D spatial planes to generate the projection images.

These 3D displacement vectors were then projected onto the XY, YZ, and ZX 2D planes. If the vectors are projected onto these planes for the entire timeframe of the sign, the projections form a 2D path that captures the pattern of the sign in the 3 planes as shown in Fig. 4. No matter at which speed the gesture was performed, these 2D projections of the gesture always provide similar patterns. Hence the temporal dependency is eliminated in this process.

After capturing the pattern of a particular gesture, we normalize the projections using the maximum and minimum values along axes. In this way, the projection from different signers results in a pattern that is similar regardless of their hand size.

The projections were generated using the Python Matplotlib^62^ library where the components of the displacement were calculated along the 3 axes and they were plotted 2 at a time for the three-axis planes (XY, YZ, and ZX). We used the line plot for this with the “linewidth” parameter set to 7 and the color of the line set to black. This resulted in 3 grayscale images for the 3 projection planes for each gesture. The images were then resized to 224 × 224 pixels dimensions and we used these images for the input of our proposed model.

### The proposed architecture

**Figure 5:**
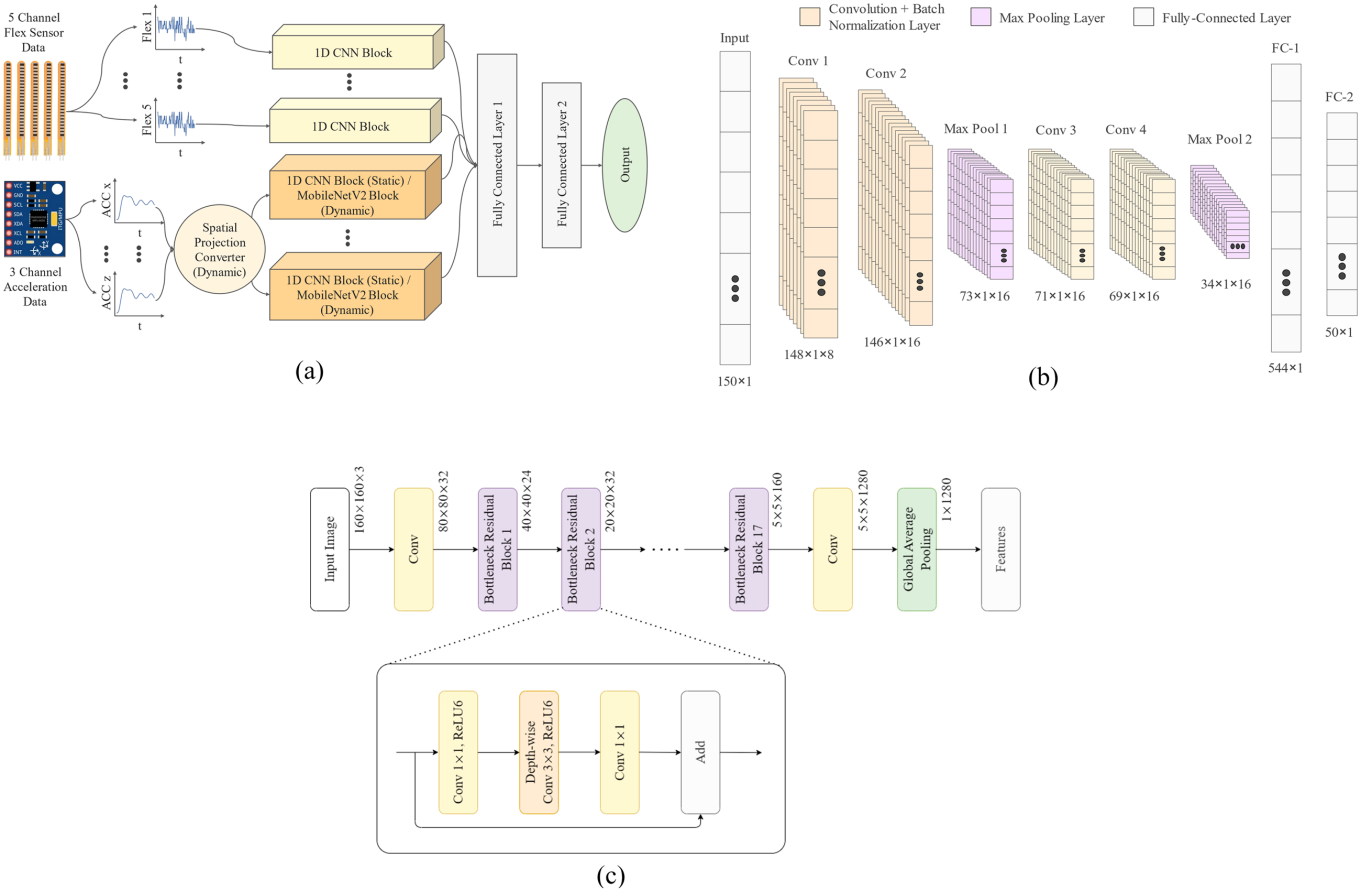
Proposed network architectures: (**a**) Overall diagram of the proposed architecture. For static gestures, the sensor channels are processed by parallel 1D ConvNet blocks. For dynamic gestures, the accelerations are first converted into spatial projection images and features are extracted from them using the pre-trained MobileNetV2 network, (**b**) the architecture of the 1D ConvNet Blocks, and (**c**) the architecture of MobileNetV2.

In this section, we present the network architecture of our proposed framework (Fig. 5). We have used two variations of the architecture for static and dynamic signs.

#### Architecture for static gestures

As mentioned in the Data Preprocessing subsection, Spatial Projection Images are not used for static gestures. The normalized time series channels are passed to separate 1D ConvNet blocks to produce embeddings. These embeddings are afterward concatenated in a fully connected layer which in turn, makes the prediction. Figure 5a shows the stacked 1D ConvNet block architecture for static gesture detection.

#### Architecture for dynamic gestures

We have utilized two different types of signals for the input to our model. First, we have the 3 spatial projection images generated from the acceleration data. Then we also have the 1D time-series signals from the flex sensors. So, in total, we have 8 channels of input data with 3 image channels and 5 time-series signal channels. Each of these channels was processed using separate ConvNet blocks to produce the embeddings from that particular channel. For the static gestures, the 8 time-series signals were processed using the parallel path ConvNet architecture shown in Fig. 5b. On the other hand, the projection images were processed by a 2D ConvNet architecture (MobileNetV2^63^) as shown in Fig. 5c. The architectural details of these two ConvNet blocks are discussed below.

#### 1D ConvNet block

The 1D ConvNet blocks are composed of 4 convolution layers. Each pair of convolution layers is followed by a BatchNormalization layer and a MaxPooling layer. The kernel size used in the convolution layers was set to 3, the stride was set to 1 and the padding was set to 1. The MaxPooling kernel size was set to 2 and the ReLU activation function was used. After the 4 convolution layers, the fully-connected layer with 50 neurons was used to extract the embeddings.

#### 2D ConvNet block

The 2D ConvNet blocks are constructed using the MobileNetV2^64^ architecture. MobileNet is an efficient architecture for mobile and embedded vision applications. It utilizes depthwise separable convolutions^65^ to significantly reduce the computational burden compared to regular convolution. In depthwise separable convolution, each of the channels is processed with the convolution filters separately and the resultants are combined using a 1 × 1 pointwise convolution. This is known as factorization and it drastically reduces the computation and model size.

The MobileNetV2^63^ is the result of the improvements done to the regular MobileNet architecture. It uses an inverted residual structure^66^ where the skip connections are between the thin bottleneck layers which improves the performance compared to the classical structure. The MobileNetV2 architecture starts with a regular convolution layer with 32 filters followed by 19 residual bottleneck layers. The kernel size was set to 3 × 3 and ReLU6^64^ was used as the activation function.

We used the Tensorflow^67^ Python library to implement the proposed network. For the loss function, we used the Sparse Categorical Cross-Entropy loss. The loss was minimized using the Adam^68^ optimizer with a learning rate of 0.0001. The network was trained for a maximum of 300 epochs with an early stopping criterion set on the validation loss with a tolerance of 30 epochs.

### Ethical approval

We took written consent from all the subjects participating in the data collection process. It was mentioned in the consent form that the data will only be used for research purposes. Moreover, the dataset does not contain any personal information of the subjects but their sex and age information.

## Results

### Evaluation criteria

#### Evaluation metrics

To evaluate our architecture for the static and dynamic gestures, we adopted four evaluation criteria, namely macro-averaged precision, macro-averaged recall, macro-averaged F1, and accuracy which are described in Eqs. (10–16).

10 $${\text{Precision}}_{i} = \frac{{TP_{i} }}{{TP_{i} + FP_{i} }}$$

11 $${\text{Precision}}_{{{\text{macro}} - {\text{avg}}}} = \frac{1}{N}\mathop \sum \limits_{i = 0}^{N} Precision_{i}$$

12 $${\text{Recall}}_{i} = \frac{{TP_{i} }}{{TP_{i} + FN_{i} }}$$

13 $${\text{Recall}}_{{{\text{macro}} - {\text{avg}}}} = \frac{1}{N}\mathop \sum \limits_{i = 0}^{N} {\text{Recall}}_{i}$$

14 $$F1_{{\text{i}}} = 2\frac{{Precision_{i} *Recall_{i} }}{{Precision_{i} + Recall_{i} }}$$

15 $$F1_{macro - avg} = \frac{1}{N}\mathop \sum \limits_{i = 0}^{N} F1_{i}$$

16 $${\text{Accuracy}} = {\text{Precisio}}n_{micro - avg} = \frac{{\mathop \sum \nolimits_{i = 0}^{N} TP_{i} }}{{\mathop \sum \nolimits_{i = 0}^{N} TP_{i} + \mathop \sum \nolimits_{i = 0}^{N} FN_{i} }}$$

where $TP$, $FP$, and $FN$ denote true positive, false positive, and false negative, respectively. Moreover, the $i$ indicates the particular gesture or subject and $N$ stands for the total number of that gesture or subject. For evaluating per-gesture performance we have used the per-class precision, recall, and F1-score, and for overall reporting, we adopted the macro-average method.

#### Validation method

There are several validation techniques used for evaluating a machine-learning (ML) model. Among these techniques, we have used the leave-one-out-cross-validation (LOOCV) method to determine the performance of the architecture. LOOCV is regarded as one of the most challenging validation techniques because for each training and evaluation session, the model is exposed to a single unseen subject’s data. Hence, if that particular subject’s data contains significant variation from other subjects in the training set, the resultant matrices are heavily penalized. Increasing the number of subjects in the training set also increases the chance of having more representative data in the test set.

However, our rationales behind using the LOOCV technique are to challenge the generalization of our trained model and test the model’s capability on unseen subject data. Here, we have separated one subject from the dataset as the test set and used the rest of the subject data as the training set. Thus, we repeated the process for all 25 subjects and evaluated the overall results at last.

### Experiments

#### Baseline methods

Since we have used a custom-made dataglove for this study and our dataset has not been benchmarked before, two classical ML and one deep learning model are employed to generate the overall result. These two classical ML algorithms provided the top performance for our previous study with the same dataglove. Moreover, 1D CNN is one of the most widely used deep learning algorithms with time-series data. Wen et al.^49^ used this architecture as the AI algorithm for their study. Hence, we chose these methods for the baseline determination. Table 1 shows the results of these baseline methods for both static and dynamic gestures.

**Table 1:** Overall evaluation of different classical machine learning and deep learning methods.

Method	Gesture type	Precision	Recall	F1	Accuracy
Logistic regression	Static	0.8128	0.8132	0.8128	0.8119
	Dynamic	0.9420	0.9405	0.9406	0.9405
Random forest	Static	0.8134	0.8125	0.8106	0.8125
	Dynamic	0.9417	0.9405	0.9407	0.9405
1D CNN	Static	0.8110	0.8111	0.8110	0.8102
	Dynamic	0.8958	0.8927	0.8934	0.8928

#### Performance evaluation of the proposed method

**Figure 6:**
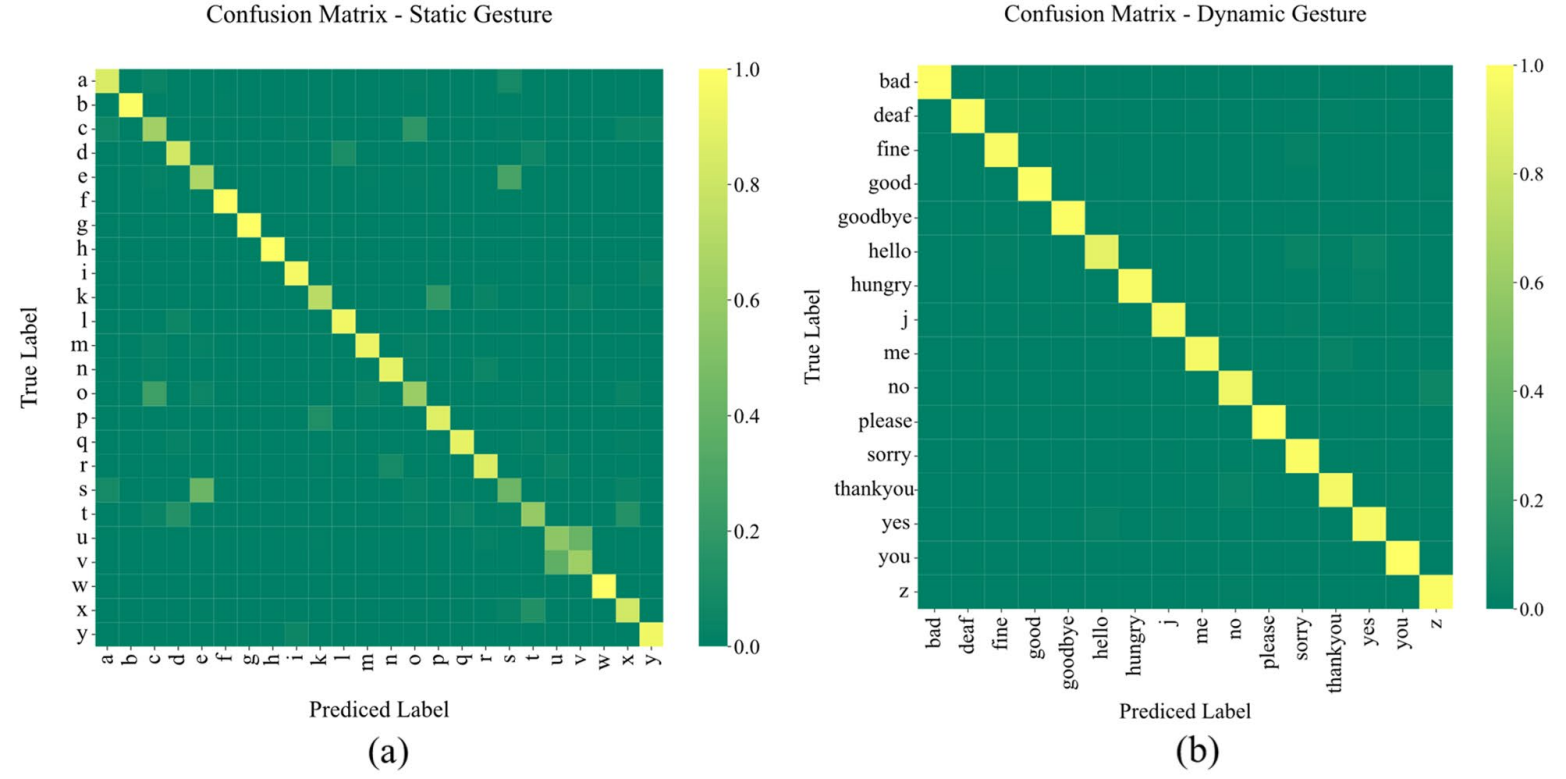
Confusion matrices: (**a**) confusion matrix for the static signs; (**b**) confusion matrix for the dynamic signs.

We have evaluated the proposed architecture for static and dynamic gestures separately. The confusion matrices illustrated in Fig. 6 projects the performance evaluation for each class. Moreover, Table 2 presents the evaluation metrics for each gesture per gesture category, and Table 3 shows the overall metrics for static and dynamic gestures.

**Table 2:** Separate evaluation of each static and dynamic sign using the proposed method.

Static gestures				Dynamic gestures			
Gesture	Precision	Recall	F1-score	Gesture	Precision	Recall	F1-score
a	0.8429	0.88	0.8611	Bad	1	0.996	0.998
b	1	0.996	0.998	Deaf	1	0.988	0.994
c	0.638	0.564	0.5987	Fine	0.9839	0.976	0.9799
d	0.7749	0.84	0.8061	Good	0.988	0.992	0.99
e	0.5611	0.716	0.6292	Goodbye	0.996	0.992	0.994
f	0.996	1	0.998	Hello	0.9655	0.896	0.9295
g	1	1	1	Hungry	1	0.976	0.9879
h	0.9843	1	0.9921	j	0.9798	0.968	0.9738
i	0.9368	0.948	0.9423	Me	1	0.96	0.9796
k	0.8042	0.772	0.7878	No	0.9553	0.94	0.9476
l	0.9144	0.94	0.927	Please	1	1	1
m	0.9424	0.916	0.929	Sorry	0.9286	0.988	0.9574
n	0.9102	0.932	0.9209	Thankyou	0.9484	0.956	0.9522
o	0.6518	0.644	0.6479	Yes	0.9234	0.964	0.9432
p	0.8301	0.86	0.8448	You	0.9843	1	0.9921
q	0.949	0.968	0.9584				
s	0.4639	0.36	0.4054				
t	0.7389	0.6	0.6623				
u	0.59	0.616	0.6027				
v	0.6224	0.6	0.611				
w	1	1	1				
x	0.7724	0.828	0.7992				
y	0.9035	0.936	0.9194				
z	0.9318	0.984	0.9572

**Table 3:** Overall metrics of the static and dynamic signs using the proposed method.

Gesture type	Precision	Recall	F1-score	Accuracy
Static	0.8223	0.8242	0.8219	0.8242
Dynamic	0.9741	0.9735	0.9735	0.9735

## Discussion

### Static gestsures

In the proposed architecture, we used individual 1D ConvNet blocks for each channel of the flex and IMU to produce embeddings. The flex sensors capture the finger movements whereas the orientation can be interpreted from the acceleration. The confusion matrix in Fig. 6a shows the majority of the detection at the diagonal with a few misclassifications. Among the 24 static gestures, 14 were classified with F1-scores over 0.8, two (k, x) had F1-scores between 0.7 and 0.8, and the F1-scores dropped below 0.7 for seven static gestures (c, e, o, s, t, u, v).

**Figure 7:**
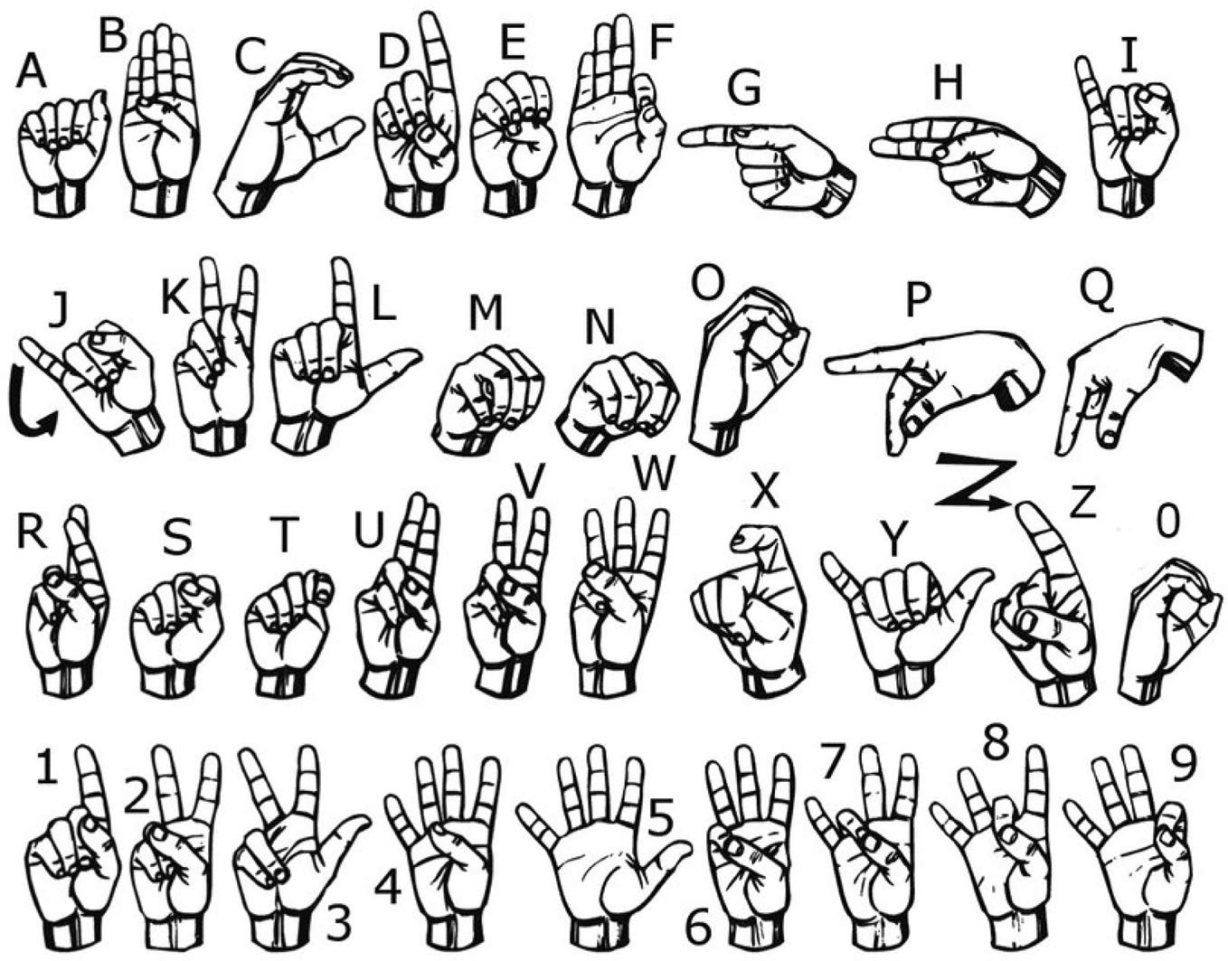
The standard ASL signs for letters and numbers^69^.

According to Fig. 7c and o are very similar to each other in gesture shape and hand orientation^69^. The only difference is the position of the thumb with respect to the other four fingers, which touch each other during o but remain separate during c. The use of a contact sensor on the tip of the thumb might improve this classification.

Moreover, *u* and *v* have similar finger flexion and orientation. The only subtle difference between these two gestures is that the index touches the middle finger during *u* but does not do so during *v*. A contact sensor between these two fingers might improve the detection ability of the model.

Based on Fig. 7, we found similarities between *e* and *s* as well. While the thumb is kept below the other fingertips during *e*, it remains on top of the fingers like a fist during *s*. Although the flexion of the four fingers is a bit different, the subtle differences in the flex sensor data are not learned by the model.

Lastly, the performance of *t* is one of the most complex ones using a dataglove where the gesture is performed with the thumb kept in between the index and the middle fingers. The finger flexion is similar in *x* as well. Moreover, for some subjects, the index finger was not bent enough which resulted in a similar flexion as *d*. Therefore, the model sometimes misclassified *t* with *x* and *d*.

Among the 0.7–0.8 F1-score range, the model falsely predicted *x* as *t* and *k* as *p* in a few cases. This is also due to the similarities between the gestures.

### Dynamic gestures

**Figure 8:**
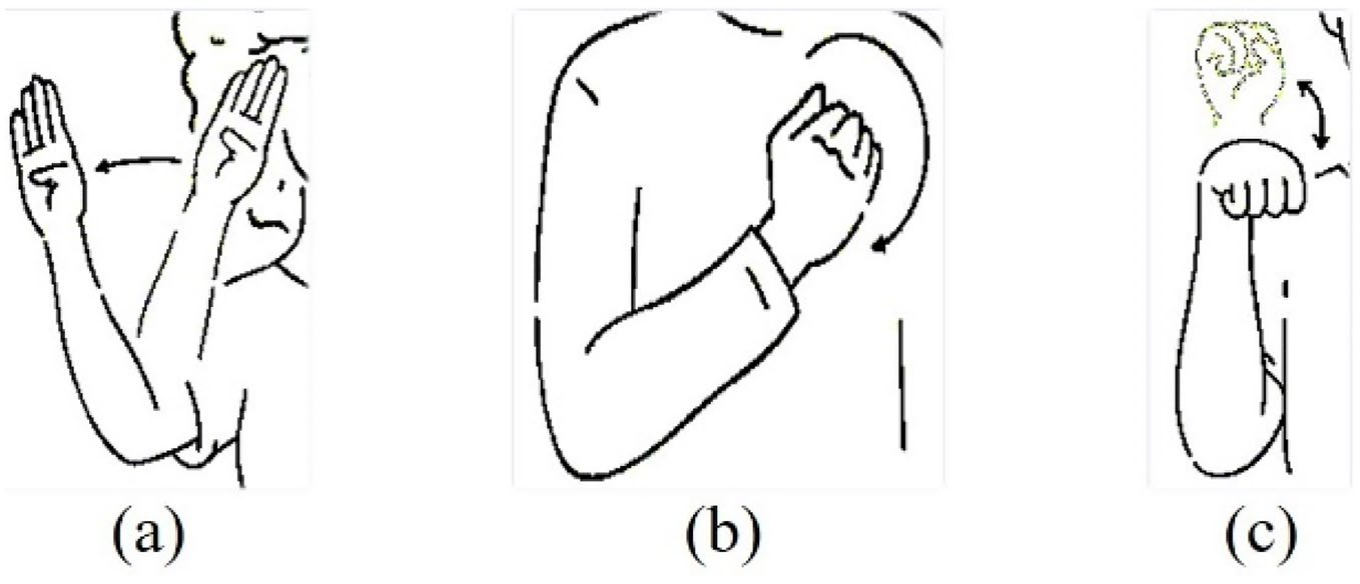
Differences among (**a**) ‘hello’, (**b**) ‘sorry’, and (**c**) ‘yes’ gestures.

Compared to the static gestures, our model performed significantly well for the dynamic gestures with an F1-score ranging from perfect 1 for *please*, to 0.9295 for *hello*. Although the gesture *hello* is significantly different from *sorry* or *yes*, according to the confusion matrix there were some misclassifications between these classes (Fig. 8 demonstrates the differences among these 3 classes). However, since we used the LOOCV technique to generate these results, the subject-induced bias in one gesture might affect the validation for a different gesture performed by another subject.

### Comparison with previous works

Based on our literature review, we showed different sensor-based gesture recognition works from 2016 in Table 4 for ease of comparison.

**Table 4:** Comparison of sensor-based hand gesture recognition works.

Author and year	Number and type of gestures	Sensor	Classifier	Number of participants	Accuracy
Faisal et al.^44^	Interaction-oriented 14 static and 3 dynamic gestures	IMU and flex sensors	KNN	30 (Static) and 5 (Dynamic)	99.53% (Static), 98.64% (Dynamic)
Wen et al.^49^	50 ASL words and 20 sentences	Triboelectric nanogenerator (TENG) sensors	1D CNN	Not mentioned	86.67%
Wang et al.^41^	51 CSL words and 60 sentences	Myo Armband (IMU, sEMG)	Multichannel CNN and attention-based encoder-decoder	34	89.2% (sentence recognition)
Saquib et al.^70^	64 ASL and BdSL alphabets	IMU, flex, and contact sensors	ANN	5	96%
Chong et al.^47^	28 ASL signs	IMU	LSTM	12	99.89%
Zhang et al.^71^	42 ASL and traffic signs	Strain sensor	DTW	3	94.58%
Yu et al.^50^	150 CSL subwords	Myo Armband (IMU, sEMG)	2D CNN and LSTM	8	95.1% (user-dependent) and 88.2% (user-dependent)
Lee et al.^45^	26 ASL alphabets	IMU, flex, and pressure sensors	Support vector machine	Not mentioned	65.7% (without pressure sensor) and 98.2% (with pressure sensor)
Jani et al.^46^	26 ASL alphabets	IMU and flex sensor	DTW and nearest mapping algorithm	8	96.50%
Abhishek et al.^72^	26 ASL alphabets and 10 letter	Capacitive touch sensor	Decision Tree	Not mentioned	92%
Wu et al.^40^	80 ASL signs	IMU and sEMG sensors	Support vector machine	4	85.24% (intra-subject) and 96.16% (combined)
Gałka et al.^48^	40 regularly used signs	Accelerometer sensor	Parallel hidden Markov models	5	99.75%
Su et al.^43^	121 CSL subwords	Accelerometer and sEMG sensors	Random Forest	5	98.25%
Savur et al.^39^	26 ASL alphabets	Myo Armband (IMU, sEMG)	Bagged Tree classifier	10	79.35%
Proposed method	**26 ASL alphabets and 14 words**	**IMU and flex sensors**	**Parallel-path CNN**	**25**	**84.42% (static) and 97.35% (dynamic)**

According to the comparison, several studies show better accuracy compared to this work. However, the number of volunteers, number of gestures, and validation method are not the same in all these studies. Moreover, due to the mode of our experiments and system, we are unable to compare our method with other systems. For example, among these works, Wen et al.^49^, Lee et al.^45^, and Abhishek et al.^72^ did not provide enough information in their manuscripts regarding the number of volunteers in their dataset. Although other works have mentioned the number of users, most of them, for example, Su et al.^43^, did not consider user-independent performance. In practice, AI-based models show discrepancies in their performances on new subjects, making the user-specific metric unreliable.

However, Wen et al., 2021^49^, Lee et al.^45^, and Saquib et al.^70^ customized their dataglove with sensor placements at some specific points to detect the touch at the fingertips. Such sensor placements have improved the detection capability of some specific ASL alphabets. In this work, we proposed a generalized hand gesture recognition system and used ASL signs only for validation. On the other hand, such ASL-specific systems in the abovementioned studies might not show similar performance in other application domains.

Moreover, the number of gestures, number of subjects, and gesture type are three significant parameters for the performance comparison. For example, in our previous work^44^, we used K-nearest neighbors (KNN) with the same dataglove which resulted in an accuracy of 99.53% for static and 98.64% for dynamic gestures. However, that study included only 14 static and 3 dynamic gestures collected from in total of 35 volunteers. However, the gestures chosen for the study were very distinct from each other compared to the ones we used in this study.

The comparison among several systems cannot be done based on only the accuracies of the systems. Based on the gesture type, number of gestures, number of volunteers, application, and validation method, this study presented a more robust and economic hand gesture recognition solution compared to the other works in recent years.

### Limitations

#### Domain-specific improvement

Each application of hand gesture recognition is different. Hence, some domain-dependent limitations are encountered in the model’s performance for a few classes which might vary for different sign language dictionaries. In this particular application, contact sensors are required at the tip of the thumb and between the index finger and the middle finger for performance improvement.

#### Limitation in everyday use

Although made using low-cost commercially available sensors and modules, the dataglove is not feasible for everyday outdoor use which limits the use of such systems in particular domains.

### Applications

#### Video conference

Due to the COVID-19 pandemic, the use of video conferences has increased in a steep curve. However, for the deaf and hard-of-hearing community, access to these video conferences is a challenge, since some platforms might not have a real-time computer vision-based sign interpreter. In this case, an accessibility software using our dataglove and proposed AI-based gesture detection system might open new avenues for the deaf and hard-of-hearing community.

#### Robot control

One of the primary applications of hand gesture recognition is controlling a remote cyber body, namely a robot using hand commands. Due to the promising performance of our dataglove and the detection algorithm, it can be a promising low-cost solution for a wide range of robot control applications.

#### Virtual reality

Nowadays, virtual reality (VR) devices are within our reach and with the announcement of Meta Verse, new avenues of VR technology have been opened. In this regard, the fundamental necessity of communicating with the cyber world is still done using wearable dataglove-based hand gestures. Our proposed dataglove can be used in conjunction with the VR headset as well.

## Conclusion

In this paper, we developed a dataglove to detect static and dynamic hand gestures and presented a novel deep learning-based to make predictions. To validate the system, we constructed a dataset of 40 ASL signs, including 24 static signs and 16 dynamic ones, from 25 subjects. For static gestures, after data filtering, we compensated the gravity from the acceleration and converted it from the body axis to the earth axis. In the case of dynamic gestures, we generated Spatial Projection Images from 1D time series acceleration data. We also introduced a parallel path neural network architecture to extract features from different sensor channels more efficiently. Our method produced better results than classical ML and CNN-based methods for both static and dynamic gestures. The achieved results are extremely promising for various applications.

In future work, we will employ our method on several applications and create a larger dataset to explore further. Moreover, by employing a multimodal technique, we can include videos with the sensor data to accumulate additional features.

## Data Availability

The datasets analyzed during the current study are available in Figshare^73^ (https://figshare.com/articles/dataset/ASL-Sensor-Dataglove-Dataset_zip/20031017).
